# Using a lamb's early-life liveweight as a predictor of carcass quality

**DOI:** 10.1016/j.animal.2020.100018

**Published:** 2021-01

**Authors:** A.G. Jones, T. Takahashi, H. Fleming, B.A. Griffith, P. Harris, M.R.F. Lee

**Affiliations:** a Rothamsted Research, North Wyke*,* Okehampton*,* Devon*, EX20 2SB,* UK; b University of Bristol*,* Bristol Veterinary School*,* Langford*,* Somerset*, BS40 5DU,* UK

**Keywords:** Conformation, Fat class, Ewe condition, Farm management, Sheep systems

## Abstract

The commercial value of lamb carcasses is primarily determined by their weight and quality, with the latter commonly quantified according to muscle coverage and fat depth. The ability to predict these quality scores early in the season could be of substantial value to sheep producers, as this would enable tailored flock management strategies for different groups of animals. Existing methods of carcass quality prediction, however, require either expensive equipment or information immediately before slaughter, leaving them unsuitable as a decision support tool for small to medium-scale enterprises. Using seven-year high-resolution data from the North Wyke Farm Platform, a system-scale grazing trial in Devon, UK, this paper investigates the feasibility of using a lamb's early-life liveweight to predict the carcass quality realised when the animal reaches the target weight. The results of multinomial regression models showed that lambs which were heavier at weaning, at 13 weeks of age, were significantly more likely to have leaner and more muscular carcasses. An economic analysis confirmed that these animals produced significantly more valuable carcasses at slaughter, even after accounting for seasonal variation in lamb price that often favours early finishers. As the majority of heavier-weaned lambs leave the flock before lighter-weaned lambs, an increase in the average weaning weight could also lead to greater pasture availability for ewes in the latter stage of the current season, and thus an enhanced ewe condition and fertility for the next season. All information combined, therefore, a stronger focus on ewes' nutrition before and during lactation was identified as a key to increase system-wide profitability.

## Implications

Improved understanding of the relationship between a lamb's early growth and the resultant carcass quality can help inform on-farm management decisions, for example flexible supplementary strategies for both ewes and lambs.

## Introduction

The commercial value of lamb carcasses is primarily determined by carcass weight and carcass quality (Rius-Vilarrasa et al. 2009). In meat markets within the European Union, the latter is most commonly represented by premiums and penalties applied according to conformation score (CS) and fat class (FC), which are visually graded by trained assessors under the EUROP classification system to differentiate products by their genuine economic value (Johansen et al. 2006). Between the two, CS characterises the desirability of carcass shape in terms of convex/concave profiles, which are known to be associated with the proportion of muscle and fat in relation to bone, and thus the quantity of saleable meat. FC, on the other hand, quantifies the amount of subcutaneous fat on the carcass visible to the assessor and is used to select a destination market with the most compatible consumer preference as well as to ensure eating quality, as carcasses which are too lean are more likely to undergo cold-shortening. While the exact scaling system varies from country to country, carcasses in the UK are graded on a 5-point scale (E/U/R/O/P) for CS and on a 7-point scale (1/2/3L/3H/4L/4H/5) for FC, yielding 35 possible combinations of outcomes at quality assessment. For CS, grade ‘E' corresponds to carcasses that are the most well-muscled and therefore the most valuable, while for FC, grade ‘1’ corresponds to carcasses that are the leanest, but not necessarily the most valuable (as explained above).

Under the EUROP system, the ability to predict carcass quality while lambs are still on farm could be of substantial value to sheep producers, as it provides opportunities for selective breeding (Jopson et al. 2004) as well as adaptive farm management (Lambe et al. 2007; Brown et al. 2015) to attract higher premiums and reduce penalties. In recent years, computer tomography (CT) (Kongsro et al. 2008) and video image analysis (VIA) (Rius-Vilarrasa et al. 2009; Einarsson et al. 2014) have both been successfully applied to predict carcass composition. Originally developed for semi-automated classification of post-slaughter carcasses, these technologies have since been extended to estimate fat and muscle densities of live animals (Clelland et al. 2014; Ibrahim 2019). However, the specialist equipment required for these analyses is costly and hence generally unsuitable for commercial producers (Jones et al. 2004).

From the practical perspective, therefore, rudimentary techniques to predict carcass quality from physical parameters of live animals may carry greater promise across a diverse range of production systems. On-farm assessment of a lamb's carcass composition is typically conducted *in vivo* by a combined method of visual appraisal and condition scoring (Stanford et al., 1998), although subjective assessment of the hind-leg shape, an easier and less time-consuming protocol, has been suggested as an alternative method for overall carcass muscularity (Wolf et al. 2006). Nonetheless, these conventional approaches are primarily designed to provide information immediately prior to slaughter, a timing too late to influence management practices for the current cohort of animals.

In contrast, animal liveweight has the potential as an informative yet easy-to-measure indicator of a wide range of animal performance traits (McAuliffe et al. 2018). It has long been established that different body tissues of livestock (organ, bone, muscle and fat) develop at different rates at each stage of physiological growth (Lonergan et al. 2019), with organ and bone maturing early, followed by muscle and finally fat. As this pattern is generally predictable and consistent, the overall shape of a lamb's growth curve has a clear impact on body composition at all ages, including carcass composition at slaughter (Hammond 1952). In other words, lambs heavier at a given age can display a different pattern of tissue development, and ultimately carcass quality, to lambs lighter at the same age even when their genetic dispositions are similar to each other. As a case in point, carcass composition of previously feed-restricted animals has a significantly higher proportion of carcass fat when compared to feed-unrestricted animals, when the former group undergoes compensatory growth to reach slaughter weight at the same age (Oddy and Sainz 2002). These findings notwithstanding, attempts to utilise such knowledge for commercial purposes have been limited to a small number of studies using mature lamb data (Stanford et al., 1998), and the relationship between a lamb's early development and final carcass grades is not currently well-understood.

The objective of the present study, therefore, was to test the hypothesis that a lamb's post-slaughter CS and FC can be predicted from liveweight information obtained at an early stage of physiological growth. As both quality scores are only observable in the form of discrete outcomes, a limited dependent variable framework was developed to estimate the probability of a young lamb subsequently realising each score and how this might change with on-farm interventions. The framework was then utilised to quantify the economic benefit of these interventions realised through increased carcass values.

## Materials and methods

### Flock management and data collection

Data used for this study were collected over seven grazing seasons from 2011 to 2017 at the North Wyke Farm Platform (NWFP: Orr et al. 2019), an instrumented sheep and cattle grazing trial based in Devon, UK (50°46′10”N, 3°54′05”W). Specifically designed for farming system-scale research, the platform comprises three self-contained enterprises (21 ha each), which operate under different sward management strategies of reseeded grass monoculture, reseeded legume/grass mix and no reseeding (permanent pasture) (Orr et al. 2016).

Lambs were produced by a mixed age flock (2–8 years) of Suffolk x Mule ewes, mated to terminal sires over a 6-week period in October and November each year. Ewes were housed over winter from the end of December, lambed indoors in March and April, and turned out to pasture with their lambs at 72 h post-lambing. During the housed period ewes were initially fed a grass silage-based ration, with multiple-bearing ewes later supplemented with concentrates for up to six weeks prior to lambing. The median lambing date recorded across seven seasons was the 30th of March, with an average birth litter size of 2.01. Ewes were checked for colostrum production immediately post-partum, and lambs from ewes providing an insufficient amount were supplemented from a donor ewe or with powdered colostrum. Lambs were reared as either singles or twins, with one of the triplet-born lambs either cross-fostered onto a single-rearing ewe (included in this study) or artificially reared (milk replacer) (not included in this study). Male lambs were castrated at 24 h post-lambing.

Once on pasture, neither ewes nor lambs received supplementary feeds. To follow the most common local practice, animals were rotated between seven paddocks within a single enterprise based on pasture cover measurements, with a target dry matter coverage of 1500–2000 kg DM/ha for the majority of the grazing season and 1800–2500 kg DM/ha during the period leading up to tupping (Penning et al. 1995). Ewes and lambs were initially placed on the same paddock during lactation and subsequently separated at 13 weeks from birth (weaning). For slaughter, lambs were initially screened by liveweight (40 kg), and those exceeding the threshold weight were then assessed for FC and ‘finish’ (muscle coverage) by being handled at the shoulder, loin, dock, rib and breast. Across seven seasons, the mean slaughter weight was 44.5 kg. The overall mortality rate of lambs was 2.58%, with 30.6% of these deaths occurring post-weaning.

The final dataset encompassed 2103 lambs, born between 2011 and 2017 to a total of 860 ewes. The liveweight of lambs was recorded at weaning and every two weeks thereafter until finishing. Cold carcass weight and carcass price for each lamb were obtained from the abattoir together with realised CS and FC. For dams, bodyweight and CS (Russel et al. 1969) were recorded at three key stages in the production cycle: tupping, lambing and weaning. Both ewes and lambs were weighed individually on a Border Software 3-way drafting weigh crate, equipped with Tru-Test MP600 load bars, a Tru-Test EziWeigh7i weighing head and a Tru-Test SRS2 stick-reader.

### Physical data analysis

For an explorative investigation of the relationship between post-slaughter CS/FC and early-life liveweight, analysis of variance (ANOVA) was initially conducted. Data were split into five groups (for CS) or seven groups (for FC) according to the realised carcass quality, and inter-group differences in liveweight were repeatedly tested using records from different timings post-weaning. This process was first implemented without fixed effects, and then duplicated by considering the potential impacts of year of production, sward type and birth litter size.

The above approach, while intuitively attractive and statistically unbiased, fails to account for the direction of causality and therefore cannot directly quantify the impact of early-life liveweight on carcass quality. To overcome this issue, corresponding multinomial logit regression models were also estimated, with the aim to quantitatively associate a lamb's early weight to the probability of the animal achieving each CS/FC category. The same set of fixed effects were included in these estimations.

### Economic analysis

To elucidate the potential financial benefit of manipulating farming systems to have different early-life liveweights, economic analysis was also carried out as part of this study. For this purpose, lambs were first allocated to three groups in equal proportions according to their weaning weights (‘light’, ‘medium’ and ‘heavy’). Realised mean carcass value within each group was then calculated using sales information received from the abattoir at the time of slaughter (current price method).

As these values are affected by price fluctuation in the market, a second set of carcass values were also calculated using a single date price for each CS/FC combination obtained from the Agriculture and Horticulture Development Board (constant price method). This process was repeated using multiple sets of price data, including those from the dates on which the 25th (early season), 50th (median) and 75th (late season) percentile lambs were slaughtered in different seasons. However, this choice was shown to have a minimal impact on inter-group variation in deadweight prices (see Table 1 for an example from the 2017 grazing season) and therefore deemed unlikely to affect inter-group variation in carcass value either. For this reason, a single set of prices, for the median-finished lamb in the most recent year (2017), was arbitrarily selected for the constant price method.

**Table 1:** Seasonal variation in sheep deadweight prices (pence/kg) during 2017.

Yearly mean		1	2	3L	3H	4L	4H	5
	E	424.6	448.5	447.5	430.6	411.2	387.9	350.2
	U	423.8	442.4	441.5	429.6	408.0	384.5	352.6
	R	413.7	432.1	431.0	424.7	409.7	386.1	353.8
	O	378.8	413.1	417.4	414.8	410.1	389.5	331.0
	P	295.1	303.7	298.4	287.5	–	–	–
								
1st quartile slaughtered lamb		1	2	3L	3H	4L	4H	5
	E	393.3	421.7	418.6	400.5	383.1	354.2	322.5
	U	398.3	416.0	413.0	400.9	378.4	359.3	320.0
	R	387.9	407.7	406.3	399.6	383.3	363.0	326.2
	O	350.4	393.5	399.6	390.6	383.9	363.9	325.0
	P	334.9	303.9	302.7	–	–	–	–
								
Median slaughtered lamb		1	2	3L	3H	4L	4H	5
	E	376.7	404.7	404.6	387.1	363.2	343.8	310.0
	U	375.4	396.9	395.9	384.6	361.6	337.3	304.9
	R	367.9	386.2	384.4	378.1	365.2	344.2	308.7
	O	342.2	365.9	368.1	368.7	370.1	368.7	310.0
	P	267.9	262.0	248.3	–	–	–	–
								
3rd quartile slaughtered lamb		1	2	3L	3H	4L	4H	5
	E	375.0	413.1	410.1	391.6	366.9	327.0	–
	U	380.6	404.6	401.2	388.2	362.1	335.3	293.3
	R	369.5	391.6	389.7	381.1	365.5	340.5	306.4
	O	324.1	366.1	371.0	370.0	368.6	348.2	300.0
	P	265.0	283.6	265.0	–	–	–	–

Note: Carcasses were graded under the EUROP scale as a combination of conformation score (E/U/R/O/P) and fat class (1/2/3L/3H/4L/4H/5).

The entire process was also repeated using alternative methods for light/medium/heavy grouping. As the results were again insensitive to the assumption (see Table 2 for an example), the original rule of splitting the flock into equal thirds was retained. Finally, using the dataset thus prepared, inter-group differences in carcass value (under both current and constant prices) were evaluated based on the standard *t*-test.

**Table 2:** Carcass value (pence/kg) of lambs split by different categorisation methods.

	Median slaughtered lamb	Lower quartile	Upper quartile	Annual mean	Actual value of NWFP⁎ lambs
(a) Weight categories defined by an alternative rule (25%/50%/25%)					
Light	387.4	405.8	392.2	433.2	362.9
Medium	387.1	407.1	392.1	433	369.2
Heavy	389.3	408.3	394.7	435.1	386
					
(b) Weight categories defined by the baseline rule (33%/33%/33%)					
Light	387.6	406	392.5	433.2	363.3
Medium	387.3	407.4	392.3	433.1	370.4
Heavy	388.9	407.9	394	434.4	382.1

⁎ North Wyke Farm Platform.

All statistical analyses, including those described in the previous subsection, were conducted using R version 3.5.1 (R Core Team, 2019). An additional package ‘mlogit’ (Croissant 2019) was used for multinomial logit regressions.

## Results

### Descriptive statistics of flock data

A summary of flock data used in the present study is given in Table 3. Notable differences in mean finishing age were observed across seven seasons, with a particular irregularity in 2012 and 2017. Both of these years are characterised by abnormal summer weather, either unusually wet (2012) or unusually dry (2017), resulting in limited pasture growth, slower lamb growth and thus reduced weaning weights. Although no such weather patterns were evident in 2015, the profitability of the system in this season was notably low. This phenomenon was primarily driven by market behaviour, as the UK saw the lowest deadweight prices for at least 5 years. This, in turn, caused an upward impact on slaughter weight, as lambs were finished later than usual to maximise the price benefit attained through heavier carcasses.

**Table 3:** Summary of ewe and lamb flock data.

Year	2011	2012	2013	2014	2015	2016	2017	Mean
Ewe data								
Age at lambing (years)	4.20 ± 0.134	4.49 ± 0.147	4.00 ± 0.115	3.59 ± 0.095	4.00 ± 0.073	4.75 ± 0.072	5.59 ± 0.072	4.26 ± 0.045
Birth litter size	2.00⁎	2.06 ± 0.025	2.01 ± 0.040	2.03 ± 0.048	1.96 ± 0.036	2.08 ± 0.043	1.97 ± 0.037	2.01 ± 0.014
Median lambing date	28-Mar	30-Mar	31-Mar	29-Mar	30-Mar	30-Mar	01-Apr	30-Mar
Lamb data								
Total lambs finished	274	266	235	258	338	360	372	300
Carcass value (£)	77.2 ± 0.30	65.6 ± 0.45	74.2 ± 0.63	66.3 ± 0.50	60.9 ± 0.27	74.8 ± 0.32	75.9 ± 0.28	70.8 ± 0.19
Slaughter age (days)	143 ± 1.7	198 ± 1.8	141 ± 2.3	157 ± 2.3	145 ± 2.0	165 ± 2.1	180 ± 1.9	162 ± 0.9
Birth weight (kg)	n.a.†	n.a.†	n.a.†	5.41 ± 0.06	n.a.†	5.22 ± 0.05	5.18 ± 0.05	5.26 ± 0.02
Weaning weight (kg)	35.6 ± 0.30	30.3 ± 0.27	33.5 ± 0.28	33.6 ± 0.28	34.8 ± 0.27	33.5 ± 0.27	31.5 ± 0.23	33.3 ± 0.11
Finishing weight (kg)	44.8 ± 0.15	44.7 ± 0.18	43.4 ± 0.15	44.2 ± 0.16	45.0 ± 0.12	44.7 ± 0.12	44.4 ± 0.10	44.5 ± 0.05

Mean value and standard error for each year unless stated otherwise.

⁎ Only twin-bearing ewes were selected for the initial year of the trial, hence lack of variation in litter size.

† Birth weights unavailable for 2011, 2012, 2013 and 2015.

In the UK, the most common target carcass classification for domestically consumed lambs is R3L. These criteria were achieved or exceeded — commonly defined as CS/FC combinations of R3L, U3L, E3L, R2, U2 and E2 — by 92% of lambs included in the present dataset (Table 4). It is acknowledged that CS/FC distributions shown here are not representative of the whole of the UK, where only 57% of carcasses meet the specification, as the study farm is located in a lowland area with relatively high-quality pasture and also receives a relatively high level of labour input (Takahashi et al. 2018). In this regard, the present research should be seen as a feasibility study using a single set of high-resolution single-farm data; the applicability of findings to different farming systems that have a wide range of CS/FC distributions be discussed at the end of the article.

**Table 4:** Spread of lamb carcass quality classifications.

	Fat class								
Conformation score		1	2	3L	3H	4L	4H	5	Total
	E	0 (0%)	8 (0.38%)	49 (2.33%)	10 (0.48%)	5 (0.24%)	0 (0%)	0 (0%)	72 (3.42%)
	U	0 (0%)	147 (6.99%)	415 (19.73%)	42 (2.00%)	2 (0.10%)	1 (0.05%)	1 (0.05%)	608 (28.91%)
	R	7 (0.33%)	571 (27.15%)	738 (35.09%)	45 (2.14%)	5 (0.24%)	1 (0.05%)	0 (0%)	1367 (65.01%)
	O	8 (0.38%)	34 (1.62%)	14 (0.67%)	0 (0%)	0 (0%)	0 (0%)	0 (0%)	56 (2.66%)
	P	0 (0%)	0 (0%)	0 (0%)	0 (0%)	0 (0%)	0 (0%)	0 (0%)	0 (0%)
	Total	15 (0.71%)	760 (36.14%)	1216 (57.67%)	97 (4.61%)	12 (0.57%)	2 (0.10%)	1 (0.05%)	2103 (100%)

### Physical data analysis

**Fig. 1:**
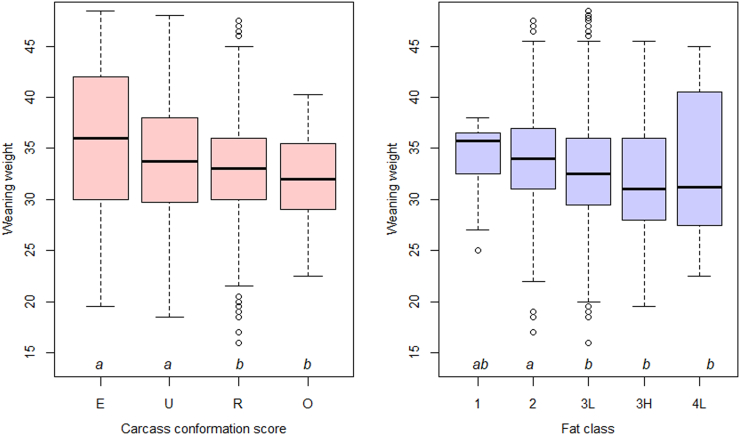
Conditional boxplots for lamb weaning weight. A significant difference in weaning weight (kg) was observed between different carcass conformation score groups (*p* < .001) (**left**) and between different fat class groups (*p* < .001) (**right**) at slaughter. Groups with the same letter are not significantly different with each other (*p* > .05) based on Tukey's honestly significant difference (HSD) test.

The results of explorative ANOVA showed a significant difference in weaning weight between CS groups (*p* < .001), with heavier animals associated with better conformation (Fig. 1a**)**. This difference was evident even after the year of production, sward type and birth litter size were accounted for as fixed effects (*p* < .001). A similar result was also observed between FC groups (*p* < .001 with and without fixed effects), with higher weaning weights associated with leaner meat (i.e. lower FC) (Fig. 1b).

Both relationships sustained when weaning (13-week) weight was replaced with 15-week liveweight, indicating the robustness of the above finding. As the season progressed, however, more animals satisfied the slaughtering criteria and thus were removed from the sample, imposing a selection bias to the dataset (**Supplementary Fig. S1**). Likely due to this change, equally strong patterns were no longer observed from data collected at 17 weeks onwards (**Supplementary Fig. S2**).

The results of multinomial regressions supported the causal relationships identified through ANOVA, with a lamb's weaning weight predicting the probability distribution for its subsequent carcass classification in a statistically significant manner. For a CS model using the score R as the baseline, an increase in weaning weight was positively associated with scores E (*p* = .008) and U (*p* = .001) (**Supplementary Table S1**). For a FC model using the score 3L as the baseline, an increase in weaning weight was positively associated with a score of 2 (*p* < .001), and negatively associated with a score of 3H (*p* = .03) (**Supplementary Table S2**). Across both models, all statistically insignificant (*p* > .05) coefficients (conformation score O and fat classes 1, 4L and 4H) were related to outcomes with low observed frequencies (Table 4).

### Economic analysis

A comparison of flock data between the three groups defined by weaning weight confirmed the expectation that lambs in ‘heavy’ group (at weaning) required a significantly shorter time to finish than ‘medium’ group lambs (*p* < .001), which, in turn, required a significantly shorter time to finish than ‘light’ group lambs (*p* < .001) (Table 5). The proportion of animals remaining on the farm after the 1st of October each year, roughly the timing when the pasture requirement for ewes increases for next reproduction, was significantly lower in the ‘heavy’ group compared to both ‘medium’ and ‘light’ groups.

**Table 5:** Economic implications of lamb weaning weight.

	Weaning weight group		
	Light (<31 kg)	Mid (>31 kg & <35 kg)	Heavy (>35 kg)
Mean slaughter age	196 days	165.3 days	129.2 days
% remaining on farm after October 1st	69.16%	27.63%	4.65%
Mean carcass value (actual price paid)	£68.48	£69.69	£73.26
Mean carcass value (at constant price)⁎	£72.96	£72.88	£74.59

⁎ As evaluated with lamb deadweight prices from 07/10/2017 (median finishing date).

There was a significant inter-group difference in the final economic value of lambs (*p* < .001 based on multi-sample *F*-test) when the current prices were applied. Carcasses from ‘heavy’ lambs were most valuable, with the average carcass value £3.57 higher than ‘medium’ lambs (*p* < .001). Carcasses from ‘light’ lambs were the least valuable, with the average value £1.21 lower than ‘medium’ lambs (*p* = .006). As the current value of a carcass reflects the seasonal variation in market price, the higher value of ‘heavy’ lambs was not only attributable to quality premium paid for improved CS/FC but also to favourable prices they attracted as a result of finishing earlier in the season.

When the effect of price fluctuation was eliminated by applying the constant price, no significant difference was observed between carcass values of ‘light’ and ‘medium’ lambs (*p* = .83). However, a significant difference remained between carcass values of ‘medium’ / ‘light’ and ‘heavy’ animals (both *p* < .001), with the ‘heavy’ group worth £1.71 more than ‘medium’ group. This result suggests that approximately half of the value difference between ‘medium’ and ‘heavy’ lambs is directly explained by physical difference in carcass quality, with the remainder indirectly through seasonal price variation.

## Discussion

### Predictability of carcass scores

The output from the multinomial models suggests that lambs which grow faster early in their lives are more likely to have leaner and more muscular carcasses when they reach the finishing weight. Availability of these predictive methods offers greater opportunities for effective flock management, where animals with either large expected premiums (for selective breeding) or large expected penalties (for adaptive management) could be segregated for bespoke grazing and supplementation strategies. To the best of our knowledge, this is the first study to quantify the impact of a lamb's early-life performance on carcass quality. However, the finding here is consistent with an already known relationship that links the stage of body growth to the composition of newly acquired tissues in domestic livestock.

Tissue development of these young animals can be simplified into four distinct phases (Lonergan et al. 2019). Shortly after birth, organs, bones and muscle all develop rapidly but with minimal fat growth (first stage). As the animal's body broadens, organ and bone approach maturity, allowing enhanced muscle development and initial formation of fat reserves (second stage). These reserves then start to increase rapidly while muscle also continues to grow (third stage). Finally, as mature weight is approached, muscle growth sharply slows down as the animal builds extra fat as energy reserves (fourth stage).

**Fig. 2:**
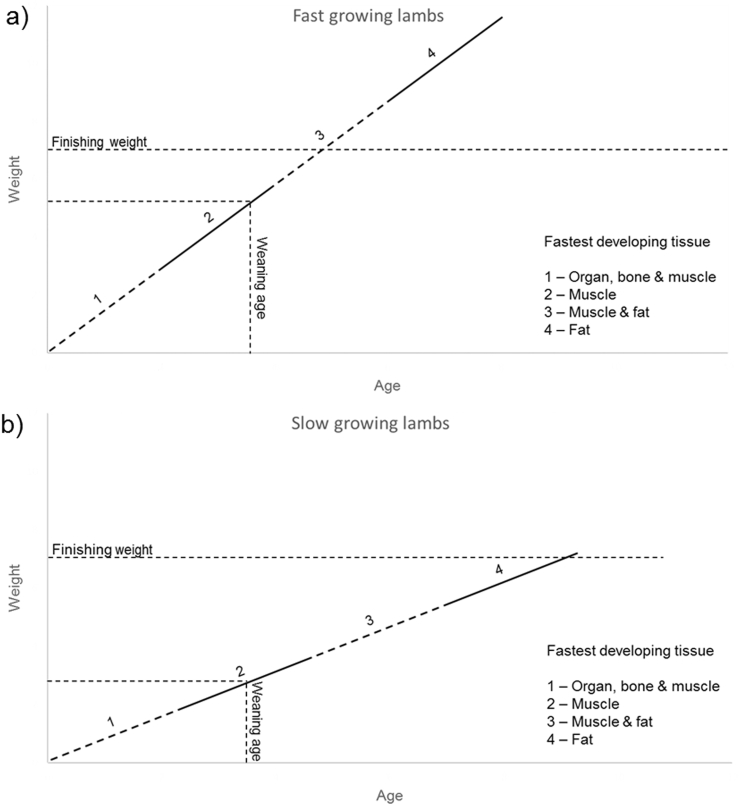
Physiological development of lambs inferred from the present study. Different tissues develop at alternate stages, with organ, bone and muscle developing rapidly in early life (1), followed by muscle (2), muscle and fat (3) and finally fat only (4) as mature weight is approached. Faster growing lambs reach finishing weight while still in an earlier stage of tissue development (**a**), resulting in a larger proportion of the carcass composed of muscle than in slower growing lambs (**b**). Straight lines are used for clarity; actual growth curves are likely to be nonlinear.

Consequently, lambs heavier at weaning are more likely to reach the target weight while still in an earlier stage of tissue development, resulting in a higher proportion of muscle and a lower proportion of fat in carcasses (Fig. 2a) compared to those lighter at weaning (Fig. 2b). A further analysis of lifetime growth data to compare ‘high-quality’ animals (eventually scoring E2) and ‘low-quality’ animals (O3L) also supports this hypothesis (**Supplementary Fig. S3**), with slopes of growth curves resembling respective conceptual representations (Fig. 2a and b**)**.

### Economic implications

It is well-established that selecting ram breeds with more favourable carcass characteristics is an effective way of improving lamb carcass quality (Jones et al. 2004; Lambe et al. 2008; Álvarez et al. 2013). However, farming systems unsuited to a change of breed or already using an optimal breed type are unable to realise this potential. In such cases, the finding from the present research may offer an alternative pathway to improve carcass quality and, in turn, system-level efficiency and profitability. Importantly, lambs heavier at weaning were more likely to result in higher-value carcasses when slaughtered, even when constant prices independent of seasonal variations were applied. This indicates that the difference in carcass values observed between different weaning weight groups was at least partially attributable to physical quality of carcasses.

To investigate the economic impact purely arising from this relationship, an auxiliary simulation was conducted using the multinomial models estimated above. For each lamb, the probability of achieving each combination of carcass scores (CS and FC) was calculated for three scenarios: actual weaning weights (baseline), baseline +6.75 kg and baseline +13.25 kg. The increments used for the latter two scenarios respectively corresponded to the interquartile range and the 90% range of weaning weights within the dataset, and thus were considered to be realistic. The derived set of probabilities was then used to calculate the expected value of the carcass for each scenario under constant prices and these values were aggregated for the entire dataset.

**Fig. 3:**
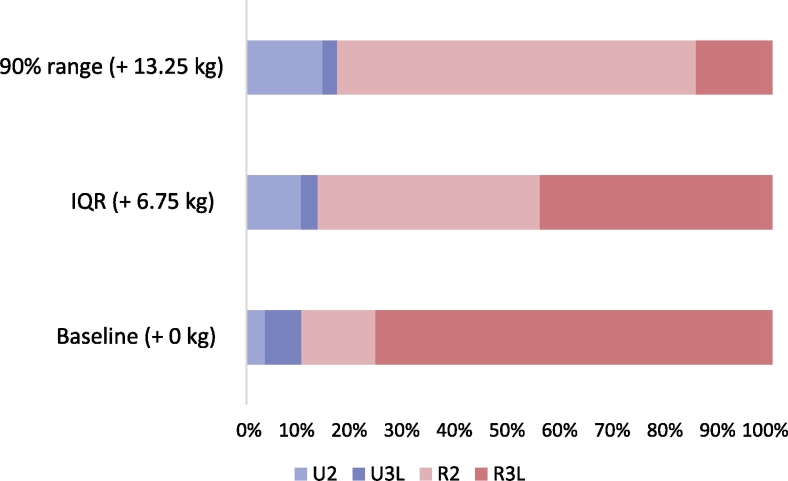
Predicted changes in lamb carcass score under enhanced weaning weight. When weaning weight becomes heavier by 13.25 kg, the likelihood of the animal attaining the fat class of 2 was found to increase dramatically (from *n* = 364 to *n* = 1708). The effect on likelihood of the animal attaining the conformation score of U, on the other hand, was only moderate (from *n* = 216 to *n* = 356). IQR: interquartile range of lamb weaning weights. U2, U3L, R2 & R3L: combined carcass quality (conformation and fat scores) under the EUROP grading system.

As expected, enhanced weaning weights were associated with an increased chance of observing higher (better) CS and lower (leaner) FC, with the second and third scenarios resulting in mean carcass values 23 and 44 pence above the baseline, respectively (Fig. 3). Across the whole dataset, the FC model was more sensitive to the weaning weight than the CS model. Nevertheless, few animals were predicted to have FC of 1, generally considered to be too lean to attract a price premium even under enhanced weaning weights. This result suggests that the risk of ‘over-fattening’ young animals is relatively low. It should be noted that the constant prices used in this model have a wider spread across CS than FC: for example, the difference in premium between FC of 2 and 3L (1.4 pence) is substantially lower than that between CS of U and R (10.4 pence). When the market places a stronger emphasis on FC, therefore, the economic impact of early life development could be greater.

In addition, achieving a higher weaning weight is likely to bring several indirect economic benefits that are not captured in the form of improved carcass quality. As alluded to above, faster growing lambs heavier at weaning are more frequently finished at an earlier point in the season and deadweight prices normally peak around the end of June — roughly the average weaning time for spring-born lambs. As can be seen in Table 5, this price fluctuation can have a considerable impact on overall carcass value, as heavy-weaned lambs were typically finished during this period of undersupply.

Faster finishing lambs are also known to be more cost efficient, regardless of carcass quality or seasonal variation in price. Assuming similar inputs per day, lambs which reach finishing age faster have lower accumulated maintenance energy and higher feed efficiency, leading to reduced feed costs (Keady and Hanrahan 2006). Even in low-input systems where pasture growth is often not directly associated with financial outlays, reduced time to slaughter is associated with lower likelihood of disease, parasitism and lameness, leading to a decrease in veterinary costs (Gascoigne and Lovatt 2015).

At the farming-systems level, there is a potential impact on pasture utilisation rate that should not be overlooked (Bohan et al. 2018). As can be seen in Table 5, less than 4% of heavy-weaned lambs were remaining on farm after the 1st of October, approximately the beginning of the next reproduction season in lowland systems, compared to nearly 70% of light-weaned lambs. Ewe nutrition is particularly crucial at this point in the season due to the association between ewe condition at tupping and fertility (Kenyon et al. 2014), and also between ewe condition at tupping and ewe condition at lambing (Gascoigne and Lovatt 2015). Ewe condition at lambing, in turn, is strongly associated with pre-weaning lamb growth in the following season (Mathias-Davies et al. 2011). Having fewer lambs remaining on the farm in the autumn, therefore, reduces resource competition and allows better pasture availability for ewes, which rear faster growing lambs with shorter finishing times and better carcass quality, to create a continuous pathway to improve the efficiency of the entire production cycle over multiple seasons. Ultimately, this change will provide an opportunity to increase the optimal stocking density — here measured by the number of breeding ewes per area — a major driver of farm-level profitability (Earle et al. 2017).

### General discussion

While the relationship between a lamb's early-life weight and carcass quality has not been previously identified, this finding does not result in producer benefit unless the animal's early-life performance can be manipulated either by selection or intervention. To this end, supplementing young lambs with creep feed is a reliable approach for improving growth rates early in their lives (Keady 2010), perhaps more so than supplementing ewes during early lactation (Campion et al. 2017). Nonetheless, the impact of such ‘forced’ growth on subsequent tissue development is not well-understood and, as animals used in this study received no supplementation and were finished entirely off pasture as part of system-scale research (McAuliffe et al. 2020), the present dataset is unable to assess this matter further. On the other hand, this research design did allow us to maintain the ‘natural’ nutrient flow from ewes to lambs, and reiterate that focussing on ewes' body condition during pregnancy may be a cost-effective way of improving lamb growth and consequently carcass value (Kenyon et al. 2014). Although outside the main scope of this study, a correlation analysis of matched data indicated a strong association between the ewe's body condition score at lambing and the pre-weaning growth rate of her lambs (*p* < .001).

Finally, it is acknowledged that all lambs from this study were of a comparable breed type, and although presenting a representative snap-shot of a typical low-land sheep enterprise in the UK, not all findings may be immediately translatable to the entire sheep industry. In particular, breed type can have a significant impact on carcass composition (Wood et al. 1980) and, in some cases, even influences the optimal stage of skeletal development for slaughter (Lambe et al. 2007). As mixed-breed enterprises are unsuitable for system-scale research with a limited number of farms, lower-resolution data from an extensive network of commercial farms are currently being analysed to investigate this issue.

## Author ORCIDs

Taro Takahashi: 0000-0002-3492-7299

## Author contributions

AJ, TT and ML designed the study. HF, BG and PH collected the data. AJ and TT analysed the data. AJ and TT prepared the draft. All authors critically reviewed the draft and contributed to the final version of the manuscript.

## Declaration of interest

The authors declare no potential conflict of interest associated with this research.

## Appendix A Supplementary data

Supplementary material Image 1
