# Supplementary material for: Using a lamb's early-life liveweight as a predictor of carcass quality
Source: Animal. 2021 Jan;15(1):None. doi: 10.1016/j.animal.2020.100018 (PMC8169456; doi:10.1016/j.animal.2020.100018)
Supplement: Supplementary file 1 — Supplementary material [file mmc1.pdf]

***animal* Journal Supplementary Material**

Using a lamb's early-life liveweight as a predictor of carcass quality

AG Jones[1,2], T Takahashi[1,2,*], H Fleming[1], BA Griffith[1], P Harris[1] and MRF Lee[1,2]

[1] *Rothamsted Research, North Wyke, Okehampton, Devon, EX20 2SB, UK*

[2] *University of Bristol, Bristol Veterinary School, Langford, Somerset, BS40 5DU, UK*

* Corresponding author: taro.takahashi@rothamsted.ac.uk

Supplementary Material (6 pages)

Table S1. Coefficients from multinomial logit regression for lamb conformation score

Table S2. Coefficients from multinomial logit regression for lamb fat class

Figure S1. Population dynamics on the farm due to removal of finished lambs

Figure S2. Relationship between lamb carcass quality measures and early-life liveweight at three different ages

Figure S3. Growth rates of lambs split by carcass quality

Table S1. Coefficients from multinomial logit regression for lamb conformation score

	E†	U†	O†
Weaning weight	0.075**	0.036**	-0.038
Grass clover lay	0.520	0.082	0.471
Perennial ryegrass lay	0.573	-0.067	-0.306
2012	-20.015	-1.243***	0.529
2013	-0.807.	-1.148***	1.342.
2014	-20.627	-1.577***	1.709*
2015	-2.117***	-0.984***	-17.792
2016	-1.637**	-0.785***	0.270
2017	-1.178*	-0.836***	0.551
Birth litter size = 1	0.824.	0.197	-0.357
Birth litter size = 3	-0.364	-0.002	-0.011
Marginal effect*	0.002	0.006	-0.002

Significance codes: *** 0.001, ** 0.01, * 0.05, . 0.1.
Fixed effect baseline variables: Permanent pasture, 2011 and Birth litter size = 1
Output baseline variable: R

* Change in average probability across the entire sample when weaning weight is increased by 1 kg from the actual value

† Carcass conformation scores under the EUROP grading system

Table S2. Coefficients from multinomial logit regression for lamb fat class

	1†	2†	3H†	4L†	4H†
Weaning weight	1.024	7.705***	-5.407*	-4.452	2.498
Grass clover lay	6.992	-3.607*	9.855*	1.263	1.712
Perennial ryegrass lay	8.921	-2.888.	3.941	-1.401	-3.060
2012	5.739	5.679**	-1.354**	-1.975	-3.192
2013	1.872	-3.125	-6.986	-1.970	-3.340
2014	2.008	7.422***	-1.750**	-2.314	-5.074
2015	1.751	-2.566	-1.446**	-2.314	-5.074
2016	1.875	-5.975	-1.078*	-1.126	-3.266
2017	1.879	1.019	-8.237.	-1.723	-5.054
Birth litter size = 1	-2.162	-1.234***	9.859**	-1.975	1.836
Birth litter size = 3	-1.118	2.436.	-2.608	-1.867	-2.011
Marginal effect*	< 0.001	0.017	-0.003	< 0.001	< 0.001

Significance codes: *** 0.001, ** 0.01, * 0.05, . 0.1.
Fixed effect baseline variables: Permanent pasture, 2011 and Birth litter size = 1
Output baseline variable: 3L

* Change in average probability across the entire sample when weaning weight is increased by 1 kg from the actual value
† Carcass fat classes under the EUROP grading system

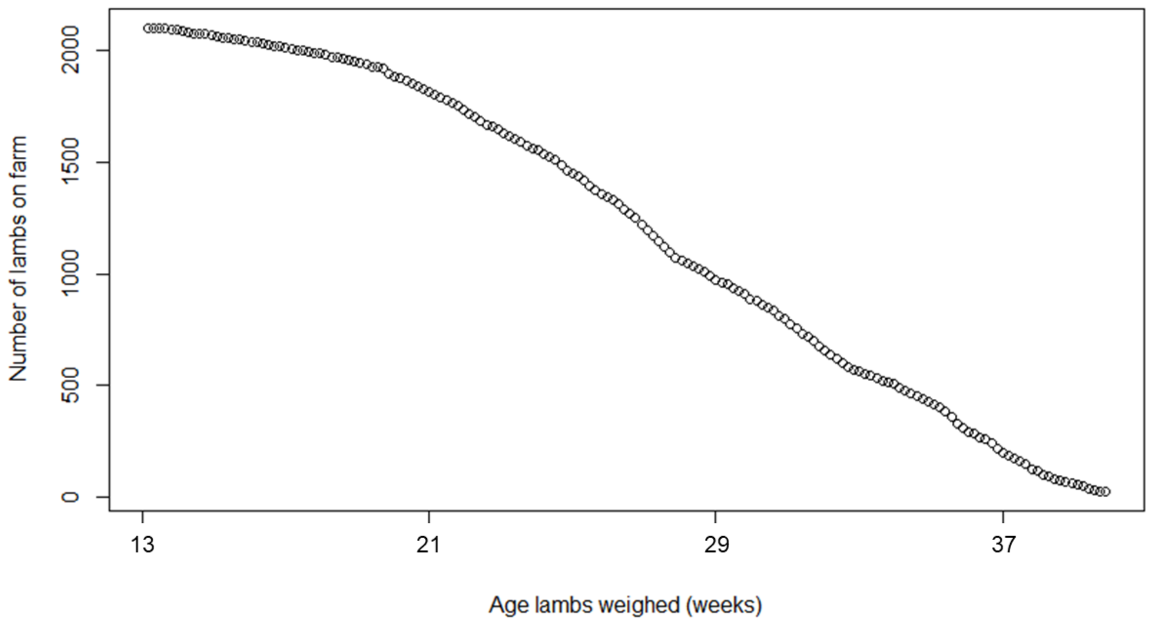

Figure S1. Population dynamics on the farm due to removal of finished lambs

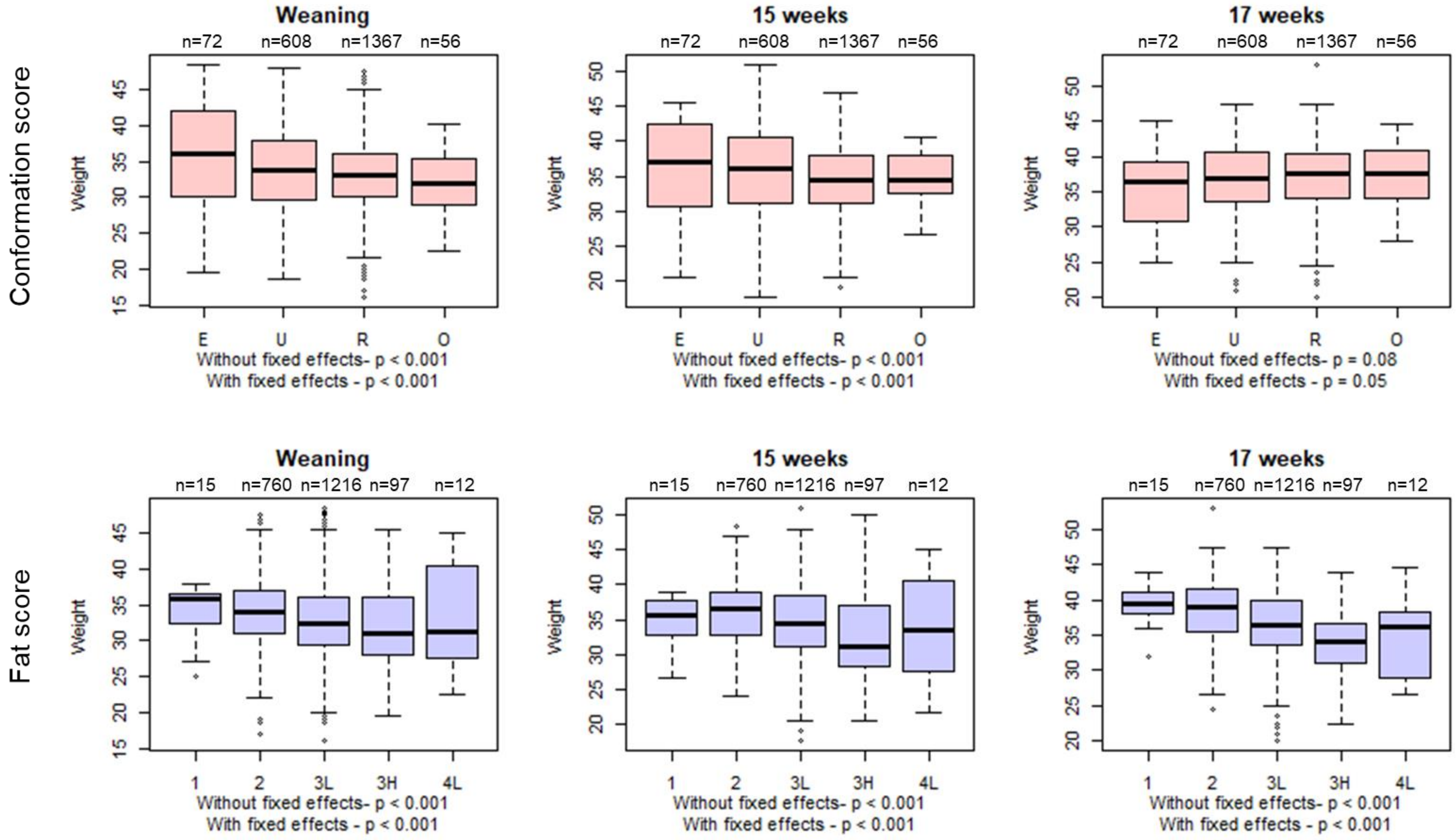

Figure S2. Relationship between lamb carcass quality measures and early-life liveweight at three different ages

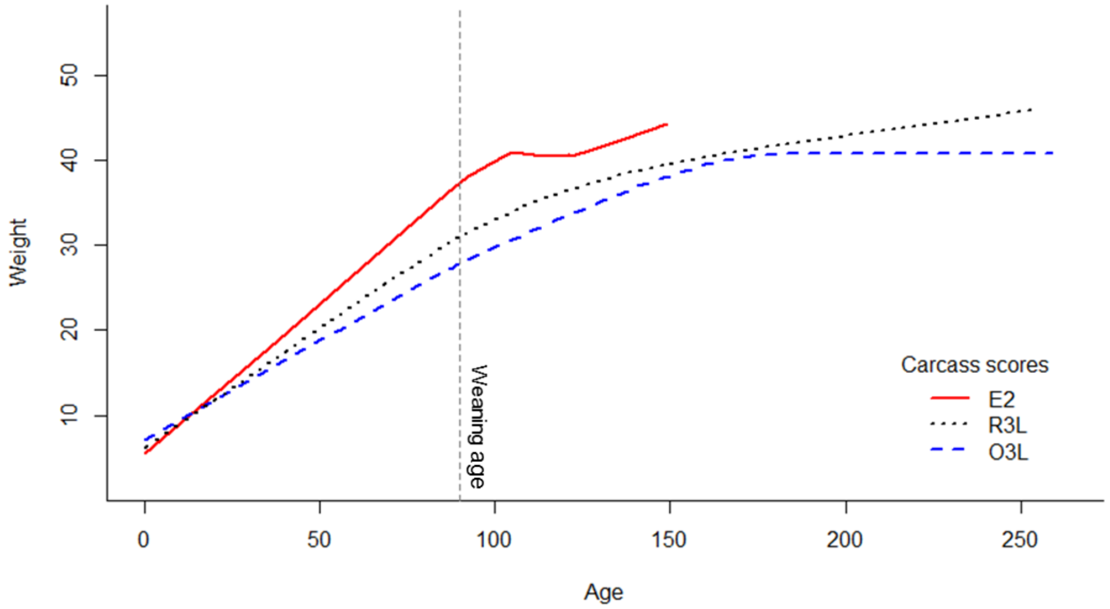

Figure S3. Growth rates of lambs split by carcass quality. These curves follow similar patterns to those predicted in **Figure 2** (main file). Lambs with high-quality carcasses (E2, *n* = 8) grew faster in early development and hence is represented by a steeper growth curve. Lambs with the most common carcass score (R3L, *n* = 728) and particularly those with low-quality carcasses (O3L, *n* = 14) were represented by flatter growth curves.
